# Measurement of Wall Shear Stress in High Speed Air Flow Using Shear-Sensitive Liquid Crystal Coating

**DOI:** 10.3390/s18051605

**Published:** 2018-05-17

**Authors:** Jisong Zhao

**Affiliations:** College of Astronautics, Nanjing University of Aeronautics and Astronautics, Nanjing 210016, China; zhaojisong@nuaa.edu.cn; Tel.: +86-182-6041-2336

**Keywords:** wall shear stress, measurement, shear-sensitive liquid crystal, jet flow, shock wave

## Abstract

Wall shear stress is an important quantity in fluid mechanics, but its measurement is a challenging task. An approach to measure wall shear stress vector distribution using shear-sensitive liquid crystal coating (SSLCC) is described. The wall shear stress distribution on the test surface beneath high speed jet flow is measured while using the proposed technique. The flow structures inside the jet flow are captured and the results agree well with the streakline pattern that was visualized using the oil-flow technique. In addition, the shock diamonds inside the supersonic jet flow are visualized clearly using SSLCC and the results are compared with the velocity contour that was measured using the particle image velocimetry (PIV) technique. The work of this paper demonstrates the application of SSLCC in the measurement/visualization of wall shear stress in high speed flow.

## 1. Introduction

Wall shear stress is an important surface quantity in fluid mechanics. In aerodynamics research, much valuable information can be gained from visualizing and measuring shear stress patterns on solid surfaces. Frictional forces that were generated by gases over these surfaces can significantly influence the performance of aircrafts. Internal frictional forces, such as those that were caused by air compression through a jet engine, also affect aircraft performance. A nice review of shear-stress measurement technique can be found in Naughton et al. [1] and Vinuesa et al. [2]. However, wall shear stress measurements remain very challenging. Although various mechanical or electrical sensors has been developed for wall shear stress measurement, such as mechanical balances, intrusive probes [1,2], or micro-electro-mechanical systems (MEMS) [3,4,5], those methods are typically complicated, disturb the flow, destroy the surface, and measure point-wise. Therefore, any means to measure wall shear stress efficiently with a high spatial resolution would be interesting.

Shear-sensitive liquid crystal coating (SSLCC) technique is a non-intrusive global wall shear stress measurement technique. The technique was first studied by Reda et al. [6,7,8,9,10] at National Aeronautics and Space Administration (NASA, Washington, DC, USA) Ames Research Center located in Moffett Field, California in the United States. The principle of this technique is to spray a thin layer of shear-sensitive liquid crystal coating onto the tested surface. The observables from the coating are transient color changes that result from the change due to the change in shear stress. The color is known to vary with the shear stress direction and magnitude, and the angles of observation and illumination. When the color is calibrated against such parameters, the visualized color images can be converted into a shear stress field. The SSLCC technique is capable of measuring the shear stress distribution over an entire surface in a continuous, non-intrusive manner. In the literature, the SSLCC technique has been used in used in several measurements including shear stress over planar surface in the flow beneath a tangential wall jet [9] and an oblique impinging jet [10], shear stress over planar surface around a cylinder in steady flow [11], and instantaneous flow [12], and even shear stress over the curved surface [13]. Extension of the SSLCC technique to the general wind tunnel measurement is available in Zhao et al. [14,15,16,17]. There are also other global wall shear stress measurement techniques, such as surface stress-sensitive film (S3F) technique [18,19], the oil-film interferometry (OFI) technique [20], the luminescent oil-film technique [21,22], the global skin friction diagnostic technique [23,24,25], and the global technique based on soft substrates [26]. The main advantage of the SSLCC technique when compared with other techniques is that the responses of SSLCC to wall shear stress are colorful and visible with a high resolution both in space, and in time, thus can immediately reveal cause-and-effect relationships between changes in model or test condition and the resulting surface shear stress field. For these reasons, SSLCC has been extensively used in flow visualization [27,28,29,30,31,32,33]. The challenge of the SSLCC technique is that the color of SSLCC is sensitive to many parameters, which make it difficult to calibrate the color. However, under some special arrangements, e.g., for normal illumination and planar test surface, some of the parameters are fixed, and thus it is easy to calibrate the color. 

There are two approaches to solve the shear stress vector from the color images of SSLCC. One is the Gauss curve fitting approach that was developed by Reda et al. [6,7,8,9], which determines the shear stress vector by fitting the SSLCC color using a Gauss curve. The other is the two-perspective approach that was originally proposed by Reda et al. [6] and further studied by Fujisawa et al. [11], in which the shear stress vector is determined by minimizing the interpolation error between two sets of calibration curves. Reda et al. [6] found that the two-perspective approach was less accurate when compared with the Gauss curve fitting approach, as it used only two observations while the Gauss curve fitting approach used five or more observations. The present paper continued the Gauss curve fitting approach by using six cameras to record the coating color at different circumferential angles synchronously, which has the potential to measure wall shear stress vector distribution in unsteady flow, while in Reda et al.’s research, a single camera was used to record the color change at different view angles, which can only be used in steady flow. Additionally, if only one camera is used, it is necessary to change the camera’s position and attitude, and to focus the lens for each view angle during the measurement. This not only increases the complexity, but it also may introduce additional noises. These shortcomings can be overcome by using several synchronous cameras.

Although some encouraging and impressive results have been obtained by the SSLCC technique under special arrangements, most wind tunnel studies of the SSLCC technique were performed in very low speed flow [11,12,13,14,15,16,17], exactly speaking, no more than 30 m/s. Some exceptions can be found in Disimile et al. [34] and Zharkova et al. [35], where only the shear magnitude field was measured. Note that the wall shear stress in low speed flow is very small and it is not easy to measure, and so there are considerable noises in the measured wall shear stresses [11,12,13,14,15,16,17]. It is believed that the shear stress that was measured using the SSLCC technique should be more accurate at higher wind tunnel velocities, because in that case the shear stresses are larger and thereby easier to measure. However, the actual performance of the SSLCC technique in high speed wind tunnel flow is unknown. This is another motivation of the present paper. In addition, significant improvement has been achieved in the digital imaging technique over the past years, which makes it is possible to measure the wall shear stress distribution accurately while using inexpensive digital cameras.

The present paper investigated the SSLCC technique in the high speed flow of a small open jet wind tunnel. Six cameras were used to record the coating color changes at different circumferential angles synchronously. The wall shear stress vector distribution over planar surface was measured and the flow structure was compared against the result that was obtained from the oil-flow technique. Effort was also made to visualize the shock waves in the supersonic jet flow using SSLCC.

## 2. Experimental Apparatus

**Figure 1:**
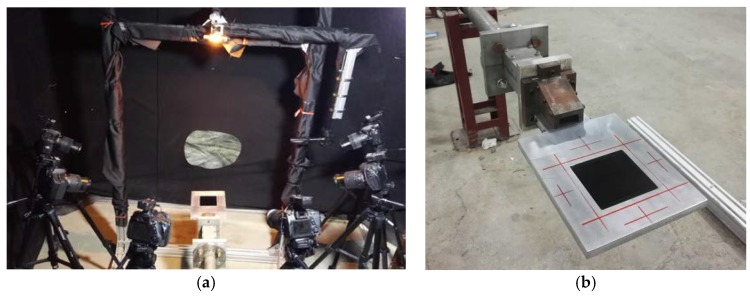
Photograph of experimental apparatus. (**a**) Overview; and, (**b**) Flat plate.

**Figure 2:**
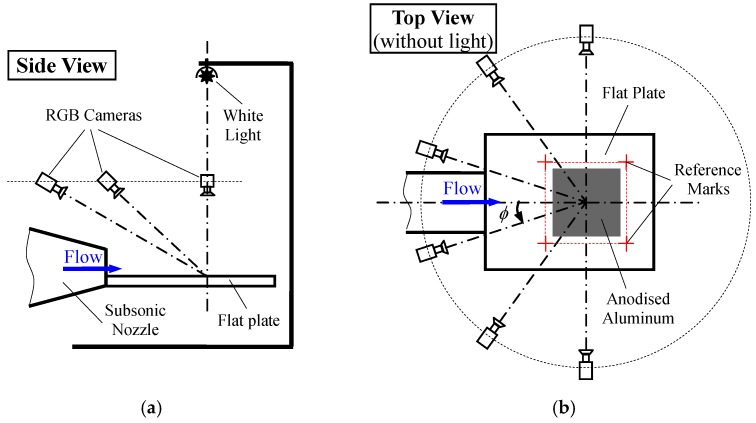
Schematic of experimental apparatus. (**a**) Side view; and, (**b**) Top view.

Measurement of wall shear stress using the SSLCC technique was carried out on a flat plate that was mounted at the mouth of a small open jet wind tunnel. Figure 1 shows two photographs of the experimental apparatus. A schematic of experimental arrangement is illustrated in Figure 2.

The size of the flat plate was 250 mm in length and 200 mm in width. A black anodized aluminum plug of 100 mm by 100 mm was mounted into the test plate in order to enhance the quality of the color change in SSLCC; with the plug leading edge 100 mm away from the flat plate leading edge. The top surface of the anodized aluminum plug was used as the test surface. A subsonic convergent nozzle was used to supply dry filtered air at room temperature from a pressurized gas tank. The nozzle pressure ratio (NPR = jet total pressure/ambient static pressure) could be regulated from 1.0 to as high as more than 6.0. The size of the nozzle exit was 4 cm in width and 2 cm in height.

A small halogen tungsten light bulb (20 W) with an aluminum cap was used as the illumination source. The light bulb was placed normally above the anodized aluminum plug, with a distance of 120 cm to reduce the light angle difference across the test surface. The characteristics of this illumination source were discussed in Zhao et al. [16]. The maximum difference of the light angles across test plug (10 cm × 10 cm) was less than 8°, which had little effect on the SSLCC color change, according to Wilder et al.’s findings [36]. It should be noted that Fujisawa et al. [11,12,13] used a stroboscope to provide illumination, but the measurements were confined to a small area of 3 cm × 3 cm, otherwise the error that was induced by the light angle difference was large. A small black room made was built to reduce the interference of the light that was reflected by neighboring objects. One drawback of this illumination source was its low intensity. As a result, the camera required a relative long exposure time, and it therefore did not enable instantaneous flow measurements, as discussed latter. The illumination source could not be simply replaced with a high intensity one because most illumination sources of high intensity usually have a large size, and thus provide complex light directions, which are not suitable for the SSLCC technique [16].

The imaging systems consisting of six synchronous cameras were used to record the colors of SSLCC. The six cameras were uniformly placed between *φ* = −90° and *φ* = 90°, where *φ* was the circumferential angle in the plane of the test surface. The cameras were positioned at a constant above-plane view angle to reduce the calibration difficulty. The above-plane view angle was defined as the angle between the camera lens direction and the plane of the test surface, and it measured positively upward from zero in the plane of the test surface. It was found in Zhao et al. [16] that the smaller the above-plane view angle, the more sensitive the SSLCC to shear stress. However, being too small above-plane view angle will induce considerable image distortion. In this test, the above- plane view angle was set to about 28.5° to have clear the color changes in the SSLCC, while avoiding obvious image distortion at the same time. The cameras that were used in the measurement were the Canon EOS 80D (made in Japan, 2017), and were synchronized by a parallel shutter release controller. The cameras were set in the “Manual” mode with the same lens aperture (*F* = 11), exposure time (*T* = 1/4 s) and ISO setting (ISO = 3200). The exposure time was automatically calculated by the cameras to ensure proper exposure. An 18% gray card was used to customize the white balance. The factory default settings were used for all of the other settings of the cameras. Note that the exposure time should be further reduced for measurement in unsteady flow. Actually, the synchronous cameras could work at a shooting speed as high as 250 Hz, but the intensity of the illumination source used in the test was not high enough and would lead to underexposure at so high shooting speed. 

The shear-sensitive liquid crystal mixtures that were used this study were the Hallcrest BCN/192 mixtures (www.hallcrest.com). The liquid crystal mixtures are temperature insensitive below the clearing point of 49 ± 1 °C, and change to colorless above it [37]. The running time of the small wind tunnel was several seconds for each test. This time period was too short for the wall temperature to change much, and so the wall temperature was below the liquid crystal clearing temperature. The liquid crystal mixtures were uniformly sprayed onto the black anodized aluminum plug with an air brush after being mixed with acetone. The acetone evaporated quickly after being sprayed, leaving a red coating on the test surface. The thickness of the coating was about 15 μm, which was measured based on mass conservation and estimated spray losses.

The experimental details that are described above for the SSLCC technique generally do not have a high requirement on the user ability/training. However, careful operation or even some practices are needed for new users to apply a uniformly thin layer of liquid crystal coating on the test surface. The use of the automatic spraying technique is a possible way to reduce human operation error. The other issue that should be noted is that the illumination source, the camera settings, and the above-plane view angle should all be the same between measurement and calibration.

## 3. Method for Transforming Color Images to Wall Shear Stress

To analyze the color changes of the SSLCC at each physical point on the test surface, the image that was recorded in each view underwent a perspective transformation to map each image coordinate onto the physical coordinate defined on the test surface. The camera external parameters (position and attitude) were determined for each image by solving the direct linear transformation (DLT) model for the four reference marks machined at the vertices of a square around the black anodized aluminum plug (see Figure 1b and Figure 2b), using a method that was developed Zhao et al. [14]. Only the circumferential angle of the camera lens is used in this study.

For color measurements, it is more useful to represent the color in terms of hue, saturation, and intensity (HSI), rather than red, green, and blue (RGB). Hue is one of the main properties of a color, and it is related to the dominant wavelength of a color. So, the hue images were used for further analysis. The second trichromic system, which was taken from [38], was used to calculate the hue

(1) $$H=\tan^{-1}\left(\frac{\sqrt{3}\cdot (G-B)}{2R-G-B}\right)+\theta ,\;\theta =\{\begin{array}{l} 0\;2R\geq G+B\\ \pi \;2R<G+B \end{array}$$

where *H* is the hue, *H* ∈ [−90°, 270°); *R*, *G*, and *B* are the three primary colors (red, green, and blue), respectively. In this trichromic system, red is placed at 0°, green is placed at 120°, and blue is placed at 240°. 

Spatial median filters over seven pixels by seven pixels neighborhood, which corresponded to 1.4 mm by 1.4 mm in physical coordinate on the test surface, were applied to the hue images to reduce the standard deviation of the hue variation, while preserving the hue gradient. 

### 3.1. Determination of Wall Shear Stress Direction and Vector-Aligned Hue

Reda et al. [6,7,8,9] found that, under normal illumination and fixed above-plane view angle, the color (hue) of the SSLCC under the effect of shear stress could be well fitted by a Gaussian curve between *φ* = −90° and *φ* = +90° at each point on the test surface

(2) $$H(\phi )=\left[H(\phi_{\tau })-H_{VN}\right]\cdot \exp\left[-{\left(\frac{\phi -\phi_{\tau }}{\sigma }\right)}^{2}\right]+H_{VN}$$

where *H_VN_* is the hue that is observed for a circumferential angle normal to the shear vector (i.e., |*φ_τ_* − *φ*| = 90°), *σ* is the standard deviation of the Gaussian distribution, *φ_τ_* and *H*(*φ_τ_*) are the direction of the shear stress vector and the hue observed when *φ* is aligned with the vector (referred to as vector-aligned hue), respectively. The vector-aligned hue could then be converted to the shear stress magnitude via a calibration curve of vector-aligned hue versus shear stress magnitude. 

**Figure 3:**
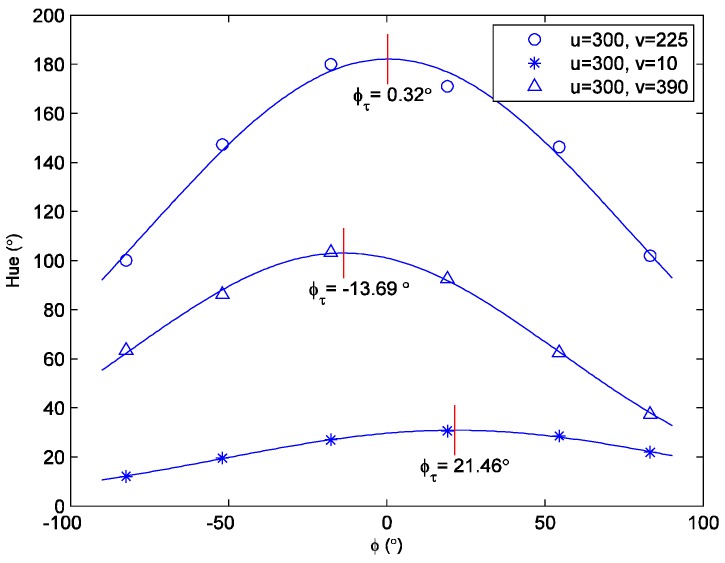
Example of hue vs. *φ* datasets at different surface points.

Three example datasets of hue versus *φ* are shown in Figure 3. The datasets were taken at three different points on the test surface (*u* and *v* are the image coordinates that will be introduced later in Section 4). The discrete datasets that are represented by different symbols (‘○’, ‘*’, and ‘△’) are the measured hue values, whereas the solid lines reflect Gaussian curve fits. The *φ* corresponding to the maximum of each curve fit is the shear stress direction *φ_τ_*. The vector-aligned hue can be converted to the shear stress magnitude via a calibration curve. For the first dataset (denoted by ‘○’), the physical point undergoing analysis was on the symmetry line of the test surface, hence the expected orientation was *φ_τ_* = 0° in this case. The shear vector orientation measured by the SSLLC technique was 0.32°. This level of agreement with a symmetry-dictated value demonstrates the accuracy of the SSLCC technique. The mean value and the standard deviation of the relative fit error for each dataset are −0.13% ± 2.24%, −0.11% ± 1.19%, and −0.16% ± 1.42%, respectively. Although no quantitative curve fit errors were given in literatures [9,10,11,12,13,14,15,16,17], it is clear to see that the noises in the curve fits in Figure 3 are much smaller than that in literatures by simply comparing Figure 3 with the hue vs. *φ* plots in the literature [9,10,11,12,13,14,15,16,17]. The improvement is attributed to the synchronous imaging system consisting of six digital cameras, the significant improvement in the imaging technique over the past two decades, and the higher shear stress levels, which are easier to measure. 

It should be noted that the above-plane view angle was fixed at 28.5° in the present research, as described in Section 2. If the above-plane view angle was decreased, then the hue-*φ* curve would extend upwards so that the hue became more sensitive to the circumferential angle *φ*, and vice versa, as reported in Zhao et al. [16]. So, for the measurement of wall shear stress distribution on the curved surface, there will be a family of hue-*φ* curves for different above-plane view angles.

### 3.2. Calibration of Vector-Aligned Hue to Wall Shear Stress Magnitude

A calibration of vector-aligned color (hue) versus shear stress magnitude is required for the specific arrangement, wherein the calibration shear stress vector is aligned with and is directed away from the camera. Reda et al. [9,10] used a variation of fringe imaging skin friction (FISF) technique, or named “oil-drop” technique, for this purpose. In the present research, we calibrated the vector-aligned hue versus shear stress magnitude using either a laminar or a turbulent friction relation over the flat plate, depending on the velocity profiles in the boundary layer. A small region that was centered about the projected nozzle centerline on the test plug was chosen as the calibration region, because the shear direction on the centerline was known in the *φ* = 0° direction. So the vector aligned color (hue) in the calibration region was recorded by a camera from the *φ* = 0° direction. Then, the only thing left to do was to determine the shear magnitude in the calibration region in order to obtain a calibration curve of vector-aligned hue and shear magnitude. It should be noted that the camera settings and the above-plane view angle should all be the same as that in the measurement. This calibration method was originally introduced in Zhao et al. [14]. 

**Figure 4:**
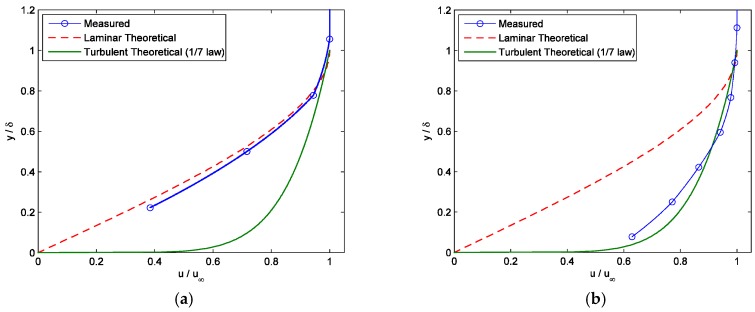
Measured velocity profile inside the boundary layer. (**a**) nozzle pressure ratio (NPR) = 1.005 (*δ* = 2.2 mm); and, (**b**) NPR = 1.026 (*δ* = 5.8 mm). (*y* is the normal distance from the test surface, *δ*_99_ is the boundary layer thickness, *u* and *u*_∞_ are the velocities inside and at the edge of the boundary layer, respectively).

The boundary layer was tripped with a turbulator (zigzag strip) at a location 9 cm upstream of the leading edge of the black plug. The height of the turbulator was 1.5 mm. The shear stress magnitude was increased by increasing the wind tunnel outlet velocity from Ma 0 to Ma 1.06 via regulating NPR from 1.0 to 2.09. The velocity profile was measured in the calibration region using a Pitot-static tube for each calibration condition to characterize the boundary layer. The boundary layer thickness was taken at the location where the mean velocity was 99% of the free stream velocity. Two examples of the measured velocity profiles are shown Figure 4, where the theoretical profiles for the laminar flow and the turbulent flow are also given for reference. Here, the 1/7 power law was used as the referenced turbulent profile for convenience. A more complete description of the mean velocity profile for the turbulent flow is available in Chauhan et al. [39]. For each calibration condition, it was easy to determine whether the special flow was laminar or turbulent by comparing the measured velocity profile against the referenced velocity profiles. It was found that the flow was laminar for NPR ≤ 1.018 (*u*_∞_ ≤ 53.4 m/s) and turbulent for NPR ≥ 1.026 (*u*_∞_ ≥ 64.2 m/s). 

For laminar flow, the skin-friction coefficient was computed from the incompressible Blasius relation,

(3) $$c_{f}=0.44\cdot {\left(Re_{\theta }\right)}^{-1}$$

where *Re_θ_* is the Reynolds number based on momentum thickness. 

For turbulent flow, the following modified Coles-Fernholz relation was used,

(4) $$c_{f}=2{\left[\frac{1}{\kappa }\ln(Re_{\theta })+C\right]}^{-2}$$

where the value of the von Kármán coefficient *κ* is 0.384 and the constant *C* takes the value 4.127. This choice of constants is due to Nagib et al. [40] and Sanmiguel Vila et al. [41], who established or verified them based on a comprehensive analysis of experimental databases. 

**Figure 5:**
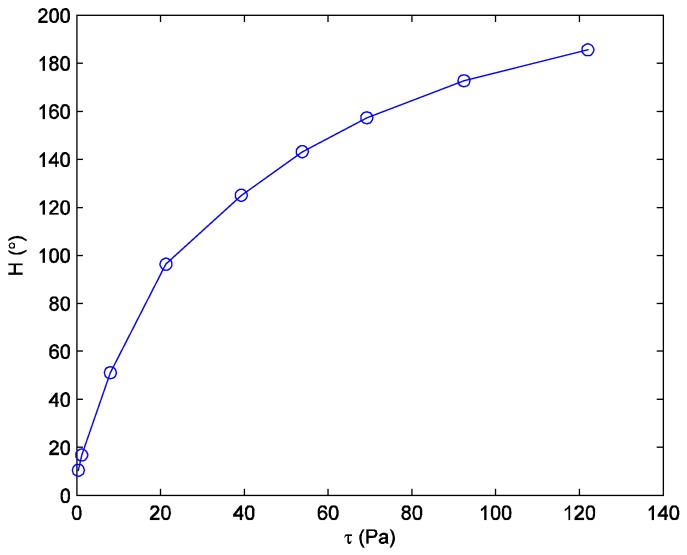
Calibration curve of hue versus shear stress magnitude for BCN/192. (*φ* = *φ_τ_* = 0°).

The momentum thickness *θ* was calculated from the measured velocity profile for each calibration condition, and then the shear magnitude in the calibration region was determined from Equations (3) or (4). Figure 5 shows a calibration curve of the vector-aligned hue versus shear magnitude that was obtained using the calibration method described above. It is seen that the vector-aligned hue increases monotonically, but not linearly with the increase of the shear magnitude. The value of the hue at the peak of the Gaussian curve fit at each physical point (see Figure 3) was then used in the *H* versus *τ* calibration curve of Figure 5 to determine the shear magnitude. It is better to perform a new calibration any time that the illumination source or the imaging system is changed.

The image processing procedure described in this section does not require special algorithms. Most steps are standard methods that are taken from literatures and are not user-dependent. The way to obtain the calibration curve may be different across users. Some more accurate ways to measure the shear magnitude should be used in future studies to ensure the calibration accuracy.

## 4. Measurement of Wall Shear Stress

**Figure 6:**
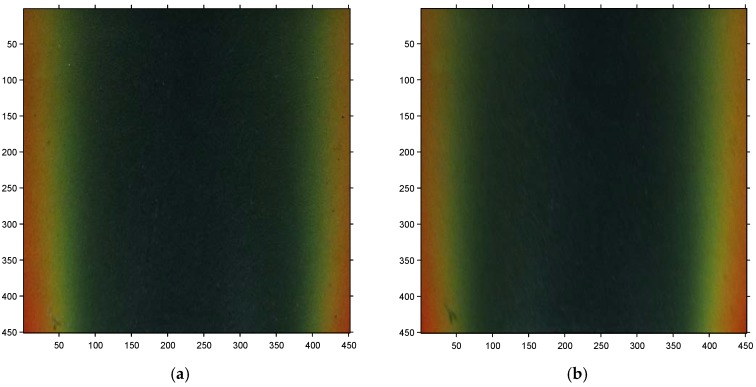

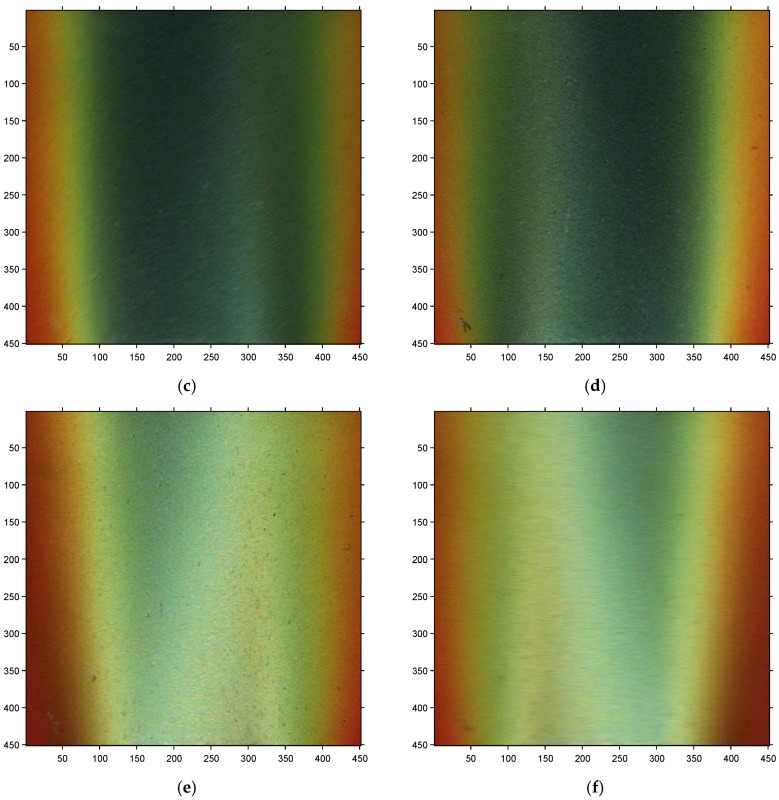
Shear-sensitive liquid crystal coating (SSLLC) color at different circumferential angles (NPR = 1.61). (**a**) *φ* = −17.7°; (**b**) *φ* = 19.1°; (**c**) *φ* = −52.0°; (**d**) *φ* = 54.5°; (**e**) *φ* = −82.4°; and, (**f**) *φ* = 83.2°. (Jet flow is from bottom to top).

In this case, the SSLCC technique was applied to the measurement of the shear stress vector distribution over the test plug beneath open jet flow. The test was carried out at NPR = 1.61 (the corresponding velocity at the nozzle exit was Mach 0.85). Six raw images that were recorded at different circumferential angles are given in Figure 6 to show the color change in SSLCC. The images were rotated into the normal view and the unwanted portions were cropped. The coordinates were in pixel units and the resolution was set so that 1 mm in physical coordinates corresponds to five pixels in the image coordinates. The centerline of each image is consistent with the projected nozzle centerline on the test surface. The main flow direction is from bottom to top. It is seen clearly in Figure 6 that SSLCC showed different color in different circumferential angles. Furthermore, the three pairs of images taken at symmetry circumferential angles are essentially mirror images, but each appears asymmetric relative to the image centerline. This asymmetry is a direct result of the dual dependence of color change response of the coating on shear magnitude and its direction relative to the observer: both quantities vary across the shear stress field.

**Figure 7:**
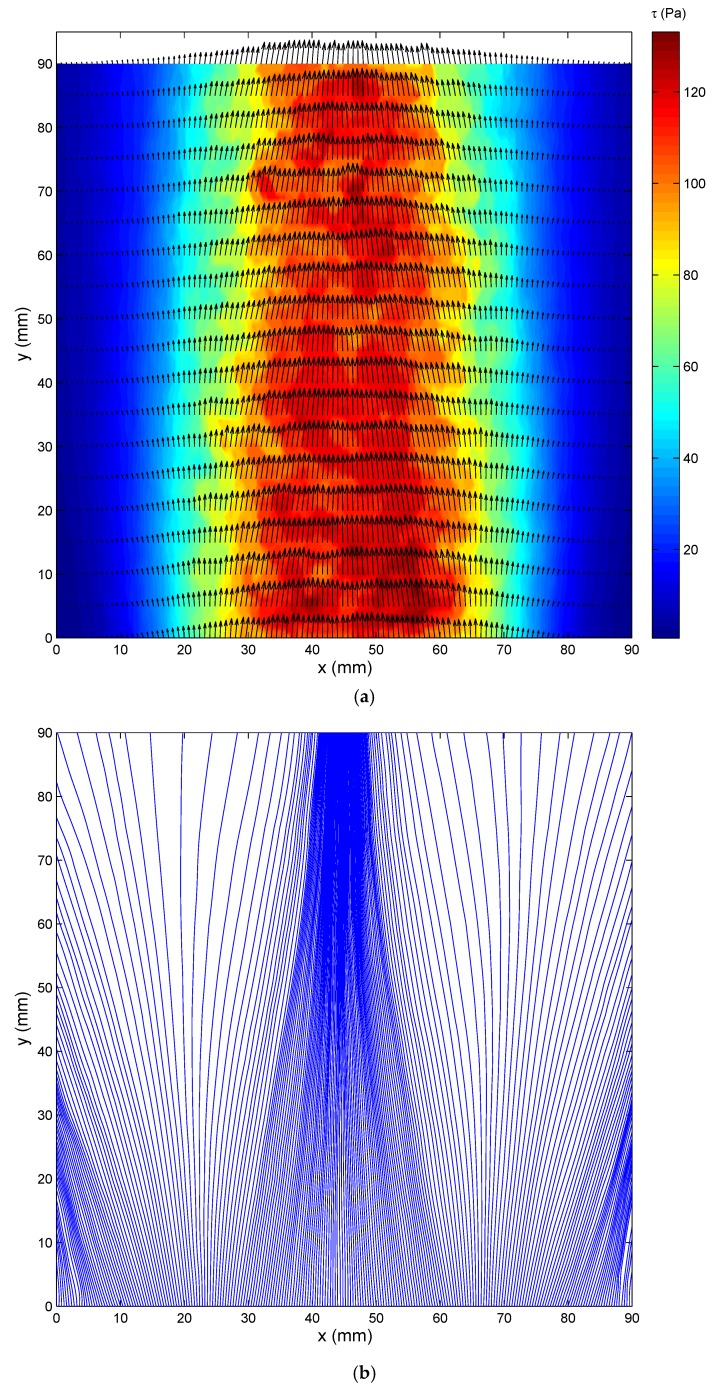
Measured wall shear stress distribution (NPR = 1.61). (**a**) Shear stress vector field; and, (**b**) Limit streamlines. (Jet flow is from bottom to top).

**Figure 8:**
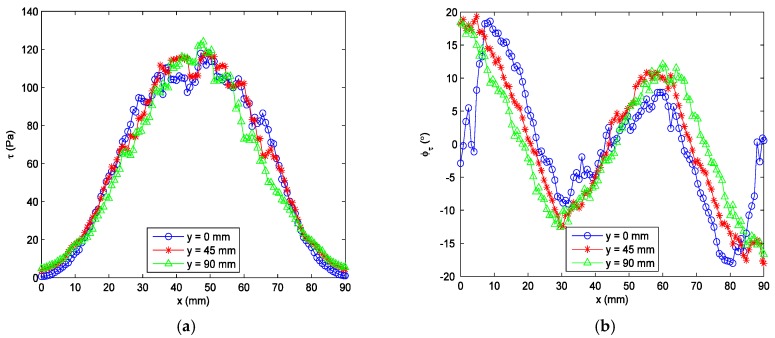
Measured shear stress vector distribution at several profiles. (**a**) Magnitudes; and, (**b**) Directions.

The curve fitting procedure that is described in Section 3 was repeated at each pixel on the test surface, and all of the shear stress vectors over the test surface could be obtained. Figure 7a shows the wall shear stress vector field that was measured by the SSLCC technique. Shear stress vectors are illustrated by vectors and are displayed every four pixels along several profiles. The figure uses false color levels to represent the shear magnitude. Figure 7b shows the limit streamlines originating from *y* = 0 and are displayed every two pixels. Each limit streamline was obtained by integrating the streamline equations in the wall shear stress vector field. It is seen in Figure 7 that the measured shear stress vector field is generally symmetrical about the centerline, although no symmetrical assumption of the jet flow was made during the measurement. This can be further verified by checking the shear magnitude and direction distributions along several *y*-constant profiles, as shown in Figure 8. The symmetrical distribution of the mean shear stress vector field is very reasonable for steady state jet flow. It should be noted that most of the fluctuations in the flow were averaged because a relative long exposure time (1/4 s) was used, otherwise, the image would be underexposed. In order to measure the fluctuations in the flow, a high intensity illumination source is needed to reduce the exposure time. In this test, the shear magnitudes vary from about 5 Pa to a maximum of about 120 Pa, and the direction vary from about −20° to 20°. The large variations in both shear stress magnitudes and directions indicate the wide sensitive region of SSLCC to shear stress vectors.

**Figure 9:**
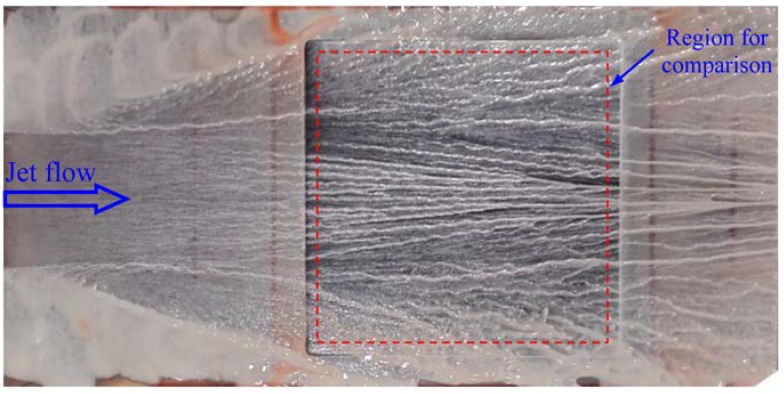
Streakline pattern on the flat plate visualized using oil-flow technique.

It is seen in Figure 7, especially in Figure 7b, that the flow converges in the center region (for 20 cm ≤ *x* ≤ 70 cm) and it diverges in the boundary regions (for *x* < 20 cm and *x* > 70 cm). This can be further verified by checking the shear stress directions given in Figure 8b. This distribution of shear stress reflects the flow structures formed as a result of the interaction between the jet flow and the ambient air. On the one hand, the static pressure in the jet flow was less than the ambient pressure because the jet flow had a high velocity. So, the flow converged under the influence of ambient pressure. On the other hand, there were induced vortexes that were generated in the free shear layer between the jet flow and the ambient air. The vortexes were counter clockwise on the left side and clockwise on the right side, and so the flow diverged in the two boundary regions outside the main flow. For comparison, the shear stress directions were visualized using the oil-flow technique. The obtained streamline pattern is given in Figure 9 and the region for comparison with the SSLCC technique is marked with a dotted box. The wall shear stress direction at a given location is believed to be tangent to the near wall streamline passing through that location. Clearly, the visualized streakline pattern confirms the shear stress vector directions that were measured using the SSLCC technique.

Quantitative comparison of the measured shear stress directions against that visualized by the oil-flow technique was not made for two reasons. First of all, the oil-flow test and the SSLCC test were not performed at exactly at the same NPR condition due to the failure of the pressure sensor. Furthermore, the oil was brushed on the surface, and its thickness was uneven and much thicker than SSLCC, which inevitably disturbed the flow. However, the quantitative error of the SSLCC-defined shear direction on the centerline was analyzed, as discussed later in the uncertainty analysis, because the shear direction on the centerline was known. Quantitative comparison of the measured shear stress vectors against the data from other techniques was not made due to the limited research conditions. Work on this topic had been made by Reda et al. [9,10], and the results showed very good overall agreement between shear vectors that were measured by the SSLCC technique and conventional point-measurement techniques (oil drop and oil flow). They found that, on average, shear magnitudes that were measured by the SSLCC technique were within 4.1% of the oil-drop data.

Uncertainties for the SSLCC-defined shear magnitudes and directions were estimated by first examining the standard deviations of the data on the projected nozzle centerline on the test surface. The centerline consisted of 451 pixels and was covered a physical length of 90 mm. The mean values and standard deviations of the shear direction on the centerline were *φ_τ_* = 0.38 ± 1.36°, while the expected shear direction on the centerline was 0° due to the symmetry of the flow. The uncertainties were also estimated by examining the standard deviations of the data within the constant-shear core region of the jet induced shear stress field. The statistics were calculated over a 3 × 20 pixel region that was centered about the centerline and having its long dimension aligned with the flow. This region covered a physical 0.6 × 4 mm extent. Within this region, the mean values and the standard deviations were *τ* = 115.5 Pa ± 0.5% and *φ_τ_* = 0.60 ± 0.64°. The standard deviations are much smaller than that obtained in low speed flow [11,12,13,14,15,16,17]. Note that the mean vector direction was only 0.60°, whereas an orientation of 0° was expected in this region due to the symmetry of the flow.

## 5. Visualization of Shock Waves

**Figure 10:**
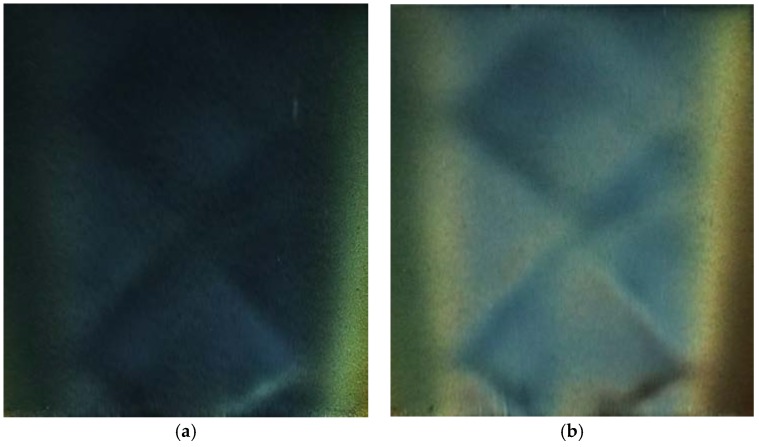
Shock diamonds visualized using SSLCC (NPR = 4.4). (**a**) *φ* = 54.6°; and, (**b**) *φ* = 84.2°. (Jet flow is from bottom to top).

This experimental test was carried out at a high NPR condition (NPR = 4.4). In this case, the under-expanded jet flow from the convergent nozzle continued to expand and accelerate in the ambient air to reach a supersonic speed. At the same time, there were shock waves formed in the supersonic flow. SSLCC was used to visualize the shear stress pattern that was induced by the supersonic jet flow. Two images that were recorded at different circumferential angles are given in Figure 10 to show the color change in SSLCC. The images were rotated into the normal view and the unwanted portions were cropped. The other two images that were recorded at the symmetrical circumferential angles are mirror images and are not shown here (no shock waves can be seen in the image recorded at about *φ* = ±18° and are either not shown here). It is in Figure 10 that the shock diamonds (also known as Mach diamonds) are clearly visualized and the shock diamonds are more clear when seen in the direction perpendicular to the main flow direction, because the flow direction changes across shock waves or expansion fans. Each shock diamond, consisting of oblique shocks and expansion fans, was formed as a result of the interaction between the supersonic jet flow and the ambient air. 

**Figure 11:**
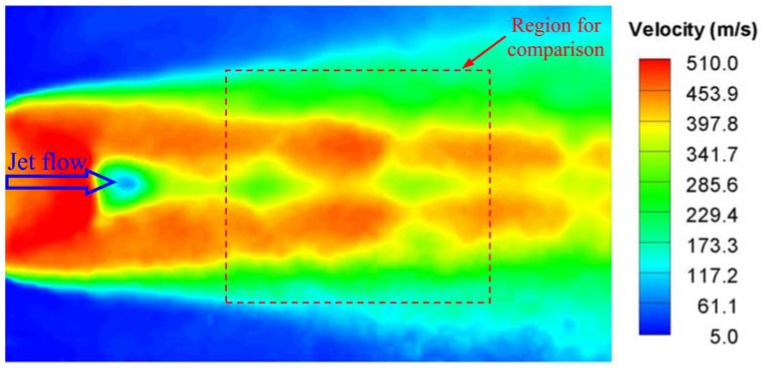
Near-wall velocity contour in supersonic jet flow measured using the particle image velocimetry (PIV) technique.

For comparison, we measured the velocity field in the supersonic jet flow using the particle image velocimetry (PIV) technique in a horizontal plane of 5 mm above the test plate to capture the near-wall characteristics. Figure 11 shows the velocity contour that was measured using the PIV technique. The PIV measurement was carried in a larger region over the entire test plate, and the region for comparison with the SSLCC flow visualization is marked with a dotted box in Figure 11. Note that the flow structures that were measured using the two techniques generally agree well with each other, but the locations of the X-shape shock waves that were visualized using SSLCC are more accurate. The present results show the potential application of SSLCC in flow visualization of shock waves.

In this test, we could not solve the shear stress field from the color images that were recorded at different circumferential angles, because the magnitude of the shear stress was too large and the SSLCC used became saturated. This can be confirmed by the dark blue that is shown in Figure 10a. However this does not mean that SSLCC cannot be used to measure the shear stress in supersonic flow with shock waves. On the one hand, the magnitude of shear stress is closely related to the dynamic pressure. In this test, the dynamic pressure of the jet flow is on the order of 100 kpa, which is larger than that in typical supersonic/hypersonic wind tunnel measurement or practical flight. Furthermore, the viscosity of SSLCC can be designed, and different viscosities are sensitive to different shear stress levels. In the literature, SSLCC have been used in flow visualization of shear stress in supersonic flows (e.g., Ma 2–4 [29]) and hypersonic flows (e.g., Ma 5 [30], Ma 8 [31], and Ma 6.85 [32]) without a saturation problem, although no visualization of shock waves has been reported.

## 6. Conclusions

The SSLCC technique is investigated in the high speed air flow of a small open jet wind tunnel. An approach to measure the wall shear stress vector distribution using shear-sensitive liquid crystal coating is established. The technique that is described in the present paper is quite simple as no special equipment or complex algorithm is needed. The wall shear stress distribution on the test surface that is induced by high speed jet flow is measured using the SSLCC technique, which captures the flow structures. Additionally, the shock diamonds inside the supersonic jet flow are clearly visualized using SSLCC. The results agree well with that obtained using the oil-flow technique or the PIV technique. Preliminary analysis shows that the uncertainties of the proposed technique in shear directions are less than 1.5°, and the uncertainties in shear magnitude are on the order of 0.5%, which are much smaller than those that were obtained in low speed flow. The results of this paper demonstrate the capability of the SSLCC technique to visualize/measure wall shear stress vector field in high speed flow. When compared with other global techniques, the advantage of the SSLCC technique is that the technique is quite simple and the responses of SSLCC to wall shear stress are colorful and visible with a high resolution both in space and in time. 

Measurement of the shear stress vector field in supersonic flow with shock waves is possible by using other shear-sensitive liquid crystal mixtures of higher viscosities. Extension of the SSLCC technique to unsteady flow requires a new illumination source of high intensity, so that the explore time of the imaging system can be further reduced. The possible problem of the SSLCC technique is that it may be limited by optical access in closed wind tunnels. The SSLCC technique that is applied through transparent test surfaces has been studied by Reda et al. [33] to solve this problem.
